# Comparative Study of Granite and Expanded Clay Aggregate as Backfill Materials for Masonry Vaults

**DOI:** 10.3390/ma17246277

**Published:** 2024-12-22

**Authors:** Piotr Krajewski, Łukasz Hojdys

**Affiliations:** Faculty of Civil Engineering, Cracow University of Technology, Warszawska 24, 31-155 Krakow, Poland

**Keywords:** arch, vault, masonry, backfill, FEM, expanded clay aggregate, ECA

## Abstract

The paper presents the results of experimental and numerical tests on barrel vaults with backfill material. The thickness, internal span, and rise of the vaults were 125 mm, 2000 mm, and 730 mm, respectively. In experimental studies, vaults with backfill of expanded clay aggregate or granite aggregate were tested. Moreover, three types of extrados finishing were considered in the experiments: masonry with flush joints, PVC film, and steel angles attached to the bricks. The numerical simulations increased the number of cases analyzed by conducting a parametric analysis for four additional backfill materials with varying bulk density or internal friction angle, as well as modifying the friction coefficient at the backfill-vault interface for each of the analyzed materials. The main goal of the analyses was to investigate the impact of the bulk density, the internal friction angle of the backfill material, and the friction coefficient between the backfill and the vault on the load-bearing capacity of the buried vault. Both the laboratory tests and numerical simulations indicate a significant impact of the internal friction angle, bulk density of the backfill materials, and the finishing method of the extrados of the vault on the load-bearing capacity of buried vaults.

## 1. Introduction

Masonry vaults are structural elements that have been used in construction for thousands of years. Although they are now built infrequently, a significant number of these vaults have survived to the present day and remain in use. Vaults were used in buildings as roofing structures and interfloors, and often served as the main load-bearing components of masonry arch bridges. Due to the geometry of vaults, it was necessary to fill the haunches of the vault with a backfill material in order to achieve a flat usable surface. Depending on the type of structure, various materials with different properties were used as a backfill material. During renovation works aimed at adapting historical buildings to modern functional requirements, it may be necessary to replace the old backfill material with new material. Additionally, when replacing the backfill, additional layers, such as thermal- or moisture-control layers, are often applied to the extrados of the vault, which alter the friction coefficient between the backfill material and the vault. This raises questions regarding which backfill material should be chosen to positively influence the load-bearing capacity of the structure and how modifications to the friction coefficient affect the behavior of the vault.

Since the 1990s, experimental research and computational analyses have been conducted to investigate the impact of the presence and properties of backfill on the load-bearing capacity of masonry arch bridges. Tests have been carried out on existing bridge structures [1,2,3,4,5], as well as on full-scale and scaled models. The studies conducted on existing bridges enabled the observation of failure modes, calibration of computational models, and validation of methods used to assess load-bearing capacity. Laboratory work provided the opportunity to analyze the impact of interactions between components of the bridge structure, as well as the effects of changes in geometry, properties of the materials used on the load-bearing capacity of the studied objects.

The research presented in [6] involved 24 models of masonry arch bridges with spans ranging from 1.00 m to 2.48 m. The primary conclusion drawn from the study was that the backfill, end walls, and wing walls increase the load-bearing capacity of arch bridge in comparison to the capacity of the standalone arch. The most significant influence of the components of the arch bridge structure on its load-bearing capacity was observed in arches with a low span-to-rise ratio, such as semi-circular arches. All models failed by the formation of four-hinged mechanisms.

In study [7], a model of a cylindrical arch bridge with a thickness of 102.5 mm and a span of 2.00 m was analyzed. The masonry arch was constructed with bricks and a cement-lime mortar, while sand was used as the backfill material. The tested specimen failed due to the formation of a four-hinged mechanism. The purpose of the study was to verify the accuracy of the methods used for assessing the load-bearing capacity of arch bridges in the United Kingdom. It was found that the assumptions in these methods are overly conservative, and the shear and normal stresses at the backfill-arch interface differ significantly from those obtained in experimental tests. This indicates that the capacity assessment methods do not adequately account for the backfill–arch interaction.

The research program described in [8] included 88 tests performed on arch bridge models with a thickness of 35 mm and a span of 700 mm, and span-to-rise ratios of 2.0 or 4.0. The arches were made with wooden elements, and the fill material was sand. The conclusions of the study indicated that the presence of backfill significantly influences the load-bearing capacity of the arch model. The failure load increased with the depth of the backfill. The lowest load-bearing capacity for the tested bridge model was observed when the load was applied at a distance from the support between 0.15 and 0.20 of the arch span.

The laboratory research program described in [9] involved 84 experiments conducted on one- and two-span arch bridge models made with wooden elements. The study analyzed the influence of backfill depth, the location of the applied load, and the bulk density of the backfill material on the load-bearing capacity of the model. It was found that the load-bearing capacity of the bridge model increased with the depth of the backfill. A significant effect of the bulk density of the fill material on the load-bearing capacity of the bridge models was also observed. The lowest load-bearing capacity for the two-span arch was recorded when the load was applied at the center of the span.

Research presented in [10,11,12] was carried out on a series of six single-span arch bridge models at a 1:6 scale. To simulate the gravitational effect in the small-scale model, tests were conducted in a centrifuge with an overload of 6× *g*. Two models, differing in the compressive strength of the arch masonry or the type of backfill material, were prepared for each case. The research results showed that the compressive strength of the arch masonry and the type of backfill material significantly affected the load-bearing capacity of the structure. The highest load-bearing capacity for the arch bridge model was obtained with backfill material of higher bulk density and internal friction angle. The use of higher compressive strength masonry led to an increase in the load-bearing capacity of the model of the arch bridge.

The research program described in [13] involved testing both small and large models of arch bridges. Twenty-seven scaled models were made from acrylic elements without mortar, and two full-scale arches were constructed from solid brick with cement-lime mortar. The backfill material for the small models was sand, while crushed limestone or clay was used for the larger models. The authors observed that when limestone was used as the backfill material, a higher failure load was achieved compared to clay. Both full-scale models failed by the formation a four-hinged mechanism. The small-scale tests were conducted to determine the influence of active and passive earth pressure of the backfill material on the load-bearing capacity of the arch bridge structure. Various backfill arrangements were considered, such as placing the fill only on the unloaded half of the arch or on the entire arch. It was found that considering passive earth pressure resulted in a 30% increase in load-bearing capacity compared to the model with no fill.

Computational analyses of arches and vaults with backfill were most commonly conducted in parallel with laboratory studies on models and tests on existing structures. These computations were performed to increase the number of cases analyzed [11], and to verify the accuracy of the assumptions underlying the introduced or existing computational methods [1,7,12,14,15,16,17,18,19,20]. In numerical studies, the behavior and load-bearing capacity of masonry arches and vaults with backfill were analyzed, along with the influence of variations in the following parameters: the internal friction angle of the backfill material [11,14,15,16,20]; the bulk density of the backfill [11,19,20,21]; the depth of the backfill [16,20,21,22]; the cohesion of the backfill [14,15]; the modulus of elasticity of the backfill and masonry [11]; the compressive strength of the masonry [11,15]. The results of the numerical analyses confirmed the observations from experimental studies. An increase in the stiffness, weight, depth, internal friction angle, and cohesion of the backfill leads to an increase in the load-bearing capacity of masonry arch structures with backfill.

The described analyses were focused on the masonry arch bridges. In the case of vaults in buildings, however, only a few publications address the issue of the interaction between the vault and the backfill [23,24,25]. When applying the recommendations based on arch-bridge research to the assessment of vaults in buildings, it is important to account for the existing structural and material differences, such as the load-bearing walls commonly found in the immediate vicinity of filled vaults, or the typically lower quality of materials used in building construction and backfill, etc. This article presents the results of experimental studies and numerical simulations conducted on models of barrel vaults with haunches filled with backfill material of geometry similar to the geometry of vaults in buildings. In the laboratory tests, expanded clay aggregate (ECA) or granite crushed stone were used as backfill materials. Moreover, three types of extrados finishing were considered in the experiments: masonry with flush joints, PVC film, and steel angles attached to the bricks. The numerical simulations increased the number of cases analyzed by conducting a parametric analysis for four additional backfill materials with varying bulk densities or internal friction angles, as well as modifying the friction coefficient at the backfill-vault interface for each of the analyzed materials. Due to the small distance between the vault and the end walls limiting the displacement of the backfill, the materials used, and the absence of dynamic interactions, the adopted model corresponds to a barrel vault in a building. The main goal of the analyses was to investigate the impact of the friction coefficient between the backfill and the vault on the load-bearing capacity of the buried vault in buildings. Additionally, the influence of bulk density and internal friction angle of the backfill material on the behavior of the analyzed structure was considered.

## 2. Materials and Methods

### 2.1. Laboratory Tests

**Figure 1:**
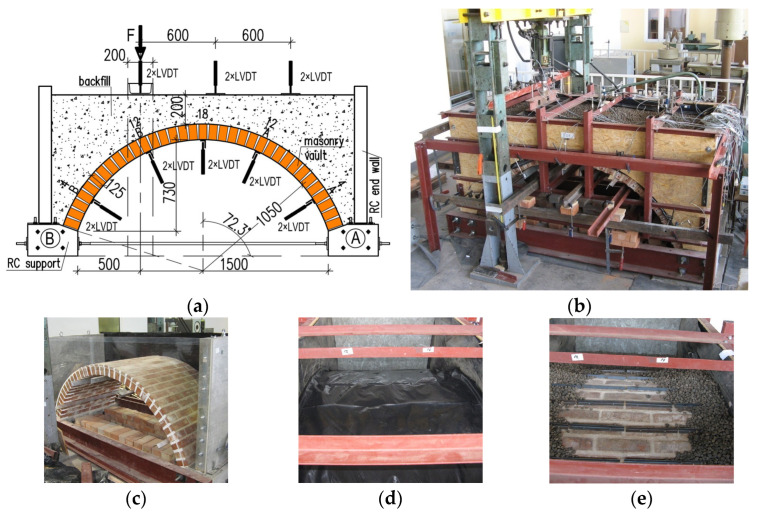
Tests on masonry vaults with backfill: (**a**) geometry of tested specimen; (**b**) test setup; (**c**) specimen with flush joints; (**d**) specimen with PVC film; (**e**) specimen with steel angles.

The main experimental investigation was conducted on seven barrel vaults of a width of 1040 mm (4 bricks), a span of 2000 mm, an internal radius of 1050 mm, a rise of 730 mm, and a thickness of 125 mm (half a brick). They were made from solid clay bricks and lime mortar. The dimensions of the tested structural elements represented a compromise between the criteria for achieving vaults with dimensions similar to those found in real-world structures and the available space in the laboratory. The vaults were supported by reinforced concrete supports connected by two steel tie rods. The tie rods prevented horizontal displacement of the supports that could have occurred during the construction or testing of the element. The geometry of the vault is shown in Figure 1a,b. The vault haunches were filled with backfill material. The thickness of the backfill layer above the crown of the vault was 20 cm. Two types of backfill materials were used in the study: expanded clay aggregate (ECA), and granite aggregate. Three different methods of finishing the vault extrados were analyzed: unplastered masonry (brickwork with flush joints), a layer of PVC film, and steel angles attached to the masonry (Figure 1c–e). Table 1 summarizes the tested elements along with their designations, extrados finishing methods, and the type of backfill material used.

The load was transferred from the hydraulic actuator to the backfill at a quarter of the span of the vault through a steel beam. During the tests, the load applied by the actuator and the displacements were measured: radial displacements of the vault, vertical displacements of the backfill, and horizontal displacements of the transverse walls. Vault displacements were measured using ten linear variable displacement transducers (LVDTs) HBM WA/50MM (HBM, London, UK) on aluminum angles fixed to the intrados of the vault. Displacements of the brick courses 4A, 4B, 12A, 12B, and 18 were measured with two sensors applied to each of the mentioned layers. Measurements on the surface of the backfill were conducted at six points (Figure 1a,b).

**Figure 2:**
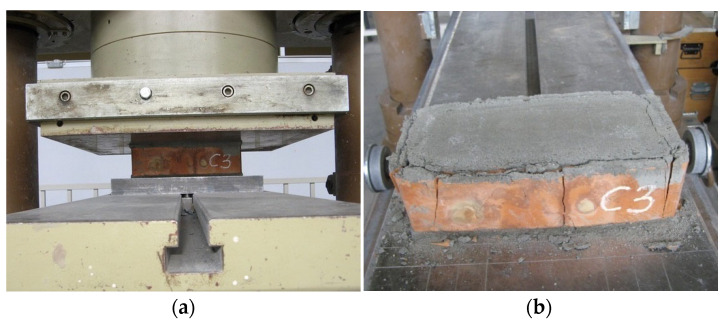
Test on compressive strength of masonry units: (**a**) specimen during testing; (**b**) view of the damaged brick.

To identify the mechanical properties of the materials used, supplementary tests were performed. The tests on the compressive strength of the masonry units were carried out on ten clay bricks according to [26] (Figure 2).

**Figure 3:**
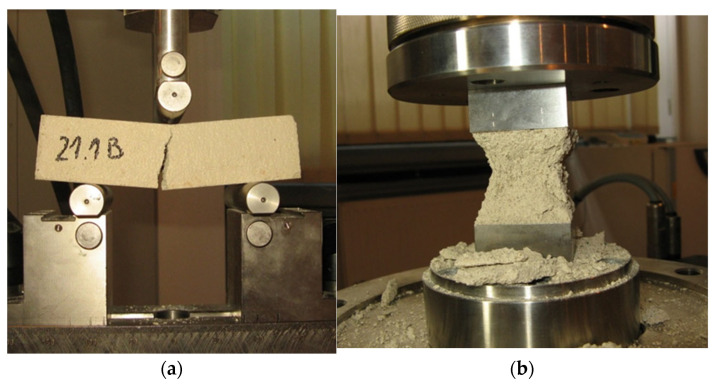
Determination of mechanical properties of mortar: (**a**) flexural tensile strength; (**b**) compressive strength.

**Figure 4:**
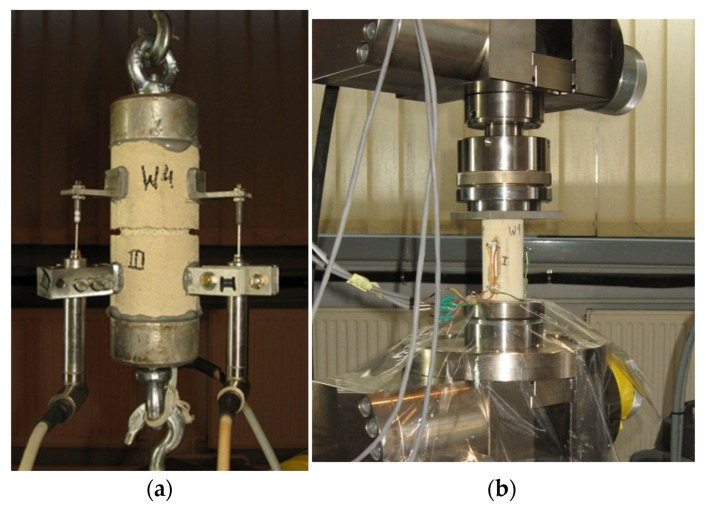
Determination of the mechanical properties of mortar: (**a**) direct tensile strength; (**b**) modulus of elasticity in compression.

For the construction of the vaults, a hydraulic lime-based (NHL-2) mortar was used. During the construction of the vaults, samples of mortar were taken from selected batches for later testing. Flexural tensile strength and compressive strength were determined on prisms/halves of prisms of dimensions 160 × 40 × 40 mm^3^ according to [27]) (Figure 3). Additionally, direct tensile strength and compressive modulus of elasticity were determined on cylindrical specimens of 50 mm diameter and 100 mm height (Figure 4). In the case of direct compressive tests, electrical resistance strain gauges of a length of 30 mm were used. The tests were carried out at 28 days.

**Figure 5:**
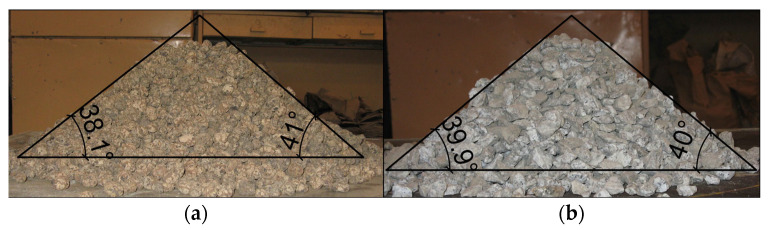
Determination of angle of repose for backfill materials: (**a**) ECA; (**b**) granite.

In the experimental campaign, two backfill materials were used: ECA and granite aggregate. For each type of aggregate, bulk density, grain size distribution, and angle of repose were determined. Bulk density was measured in a cylindrical container of a volume of 10 L. Three samples from each type of aggregate were taken for testing, in accordance with [28]. Additionally, the density of the aggregates was measured when fully compacted. The samples were mechanically compacted on a vibrating table until no volume change was recorded after one minute of compaction. The grain size distribution of the aggregates used as backfill in the experiments was determined by sieving [29,30,31]. Three samples were taken from each backfill material. The angle of repose was determined by piling the tested aggregate and measuring the angle of the slope of the cone relative to the horizontal plane. For each aggregate, 25 measurements were taken (Figure 5).

**Figure 6:**
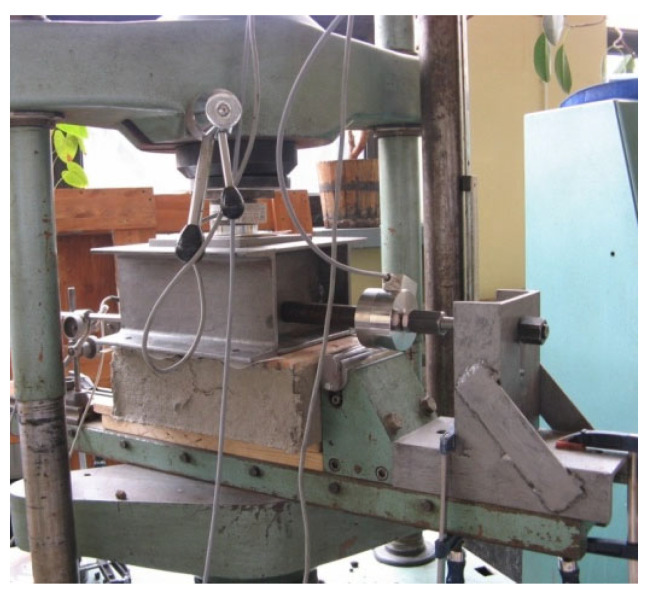
Test setup for determining friction coefficient between aggregate and masonry for different finishing of masonry surface: flush joints, PVC film, steel angles.

The friction coefficients between the aggregate (ECA or granite) and masonry were determined for three types of masonry surface finishes, as used at the extrados of the vaults: unplastered masonry (flush joints), masonry covered with PVC film, and masonry with installed steel angles. The friction coefficients were determined using a testing setup consisting of a steel box in which soil samples measuring 200 × 200 × 150 mm^3^ were placed, a masonry prism with a properly prepared contact surface, and a supporting structure allowing horizontal movement of the box. Vertical load was applied to the upper surface of the soil sample through a 25 mm thick steel pressure plate. For low normal stress levels at the contact between the backfill and masonry (below 0.02 N/mm^2^), steel weights were placed on the pressure plate, while higher stress levels were achieved by applying the load using a hydraulic testing machine. During the tests, vertical load, horizontal displacement of the box, and the horizontal force required to move the sample were recorded. Horizontal displacement was measured using two LVDTs, while vertical and horizontal forces were measured using strain gauge force transducers. A view of the test setup for determining the friction coefficient is shown in Figure 6.

### 2.2. Numerical Analysis

Due to the limited number of backfill materials and surface finishing methods tested, the study was expanded to include additional analyses using the finite element method. The primary objective of the computations was to examine the effect of the friction coefficient between the backfill and the masonry on the load-bearing capacity of the analyzed buried vault. Additionally, an attempt was made to investigate the influence of changes in bulk density and the internal friction angle of the backfill material on the ultimate failure loads.

**Figure 7:**
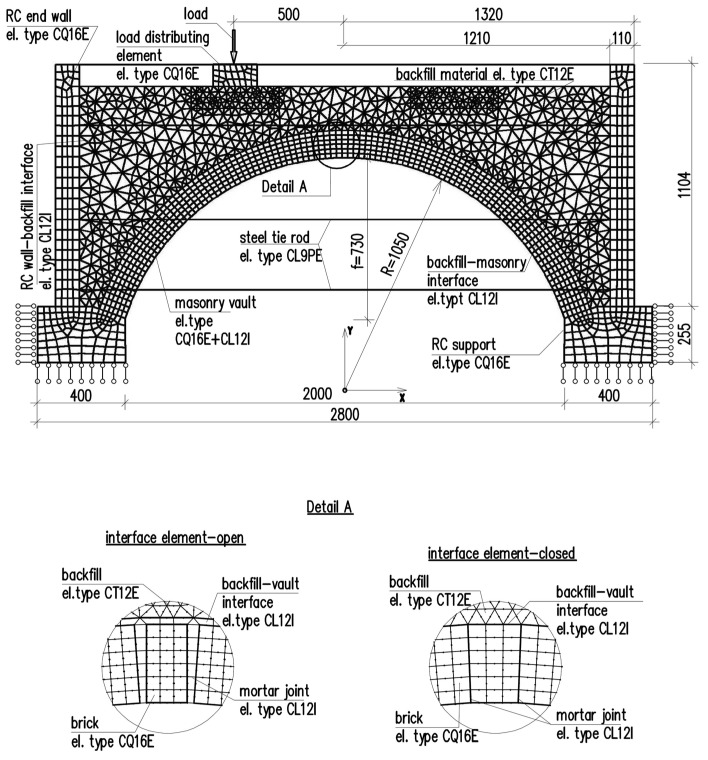
Finite element model for masonry vault with backfill—mesh topology.

The vault with backfill was modeled in a plane strain condition. The finite element model used in the analysis included the main components of the experimental setup, such as the masonry vault, reinforced concrete supports, transverse walls, and the backfill material in the haunches of the vault (Figure 7). A simplified micromodel was adopted for the masonry, consisting of linearly elastic blocks (bricks) with a Young’s modulus of 11,000 N/mm^2^ and a Poisson’s ratio of 0.2, connected by a zero-thickness interface that accounts for the mortar behavior and the brick-mortar interface properties (Combined Cracking-Shearing-Crushing interface) [32,33]. The use of this model forced the formation of damage of masonry at the locations of mortar joints, which allowed for the replication of the failure modes observed in the experimental tests of the vaults with backfill. Based on the laboratory tests and references [32,34,35], the following parameters were used for the interface in the masonry: normal stiffness of 22 N/mm^3^, shear stiffness of 10 N/mm^3^, tensile strength of 0.05 N/mm^2^, tensile fracture energy of 0.006 N/mm, a friction angle tangent of 0.75, a dilatancy angle of 0°, cohesion of 0.1 N/mm^2^, shear fracture energy of 0.01 N/mm, compressive strength of 5.5 N/mm^2^, compressive fracture energy of 1.36 N/mm, and an equivalent plastic relative displacement of 0.4.

The backfill materials used in the tests on vaults and analyses were cohesionless soils. To model such a material, an isotropic elastic-ideal plastic material model with a Mohr-Coulomb failure surface was adopted. The bulk density and internal friction angle for the analyzed backfills are summarized in Table 2. The elastic modulus for expanded clay aggregate (ECA) was taken as 15 N/mm^2^, based on the results of studies presented in [36,37,38], while for granite aggregate and four materials AM1–AM4, the Young’s modulus was assumed to be 90 N/mm^2^. Poisson’s ratio was assumed to be 0.2. The dilatancy angle was taken as 29° for ECA and 30° for the other aggregates, based on [39,40,41,42,43,44,45].

Reinforced concrete supports and transverse walls were modeled as linear-elastic, with a Young’s modulus of 30,000 N/mm^2^ and a Poisson’s ratio of 0.2.

To model the connection between the backfill and the vault, and between the backfill and the walls, interface elements with zero thickness and a Coulomb friction model were used [33]. For the backfill made of ECA, a shear stiffness of 0.03 N/mm^3^ was adopted, while for the other materials, it was set to 0.04 N/mm^3^. The normal stiffness was set to 3.0 N/mm^3^ for ECA and 4.0 N/mm^3^ for the other materials. The backfills used in the study were made of granular materials without cohesion. To ensure the stability of the calculations, a cohesion of 0.001 N/mm^2^ was assumed. The friction coefficient between the backfill material and reinforced concrete transverse walls was assumed as 0.8. To verify the adopted computational model, a comparison was made between the numerical simulation results and the experimental results from tests on vaults S03KM, S11KM, and S07GM, assuming a friction coefficient between backfill and vault of 0.8 (unplastered masonry). After verifying the model, numerical simulations were carried out for all the assumed backfill materials with various friction coefficient values at the backfill-vault interface: 0.0, 0.25, 0.5, 0.8, 1.0, 1.25, 1.5, and 2.0.

The finite element model (FEM) used for the analysis included the main components of the test setup, namely the masonry vault, reinforced concrete supports, transverse walls connected by steel tie rods, and the backfill material at extrados of the vault. In FEM model quadrilateral eight-node elements (CQ16E) for the masonry bricks and concrete elements were adopted. For the interfaces, CL12I interface elements with 3 + 3 nodes (three nodes at each line) were used. The steel tie rods were modeled using three-node line shaped plane strain elements (CL9PE). The backfill material was modeled using triangular six-node elements (CT12E) [33]. The finite element mesh was carefully chosen to ensure the convergence of the numerical model. The mesh was refined in critical areas, such as the region where the load is applied, to avoid potential convergence issues and ensure reliable results. The mesh topology for the assumed FEM model is presented in Figure 7.

## 3. Results

### 3.1. Results of Laboratory Tests

Based on laboratory tests, basic mechanical parameters of the materials used were determined. The compressive strength of the bricks, determined from the tests, was 24.4 N/mm^2^, with a coefficient of variation (CV) of 11%. The compressive and flexural strength of the mortar at 28 days were 1.1 N/mm^2^ (CV 25%) and 0.9 N/mm^2^ (CV 46%), respectively. The direct tensile strength and the compressive strength of the cylindrical samples were 0.08 N/mm^2^ (CV 21.2%) and 0.5 N/mm^2^ (CV 21.5%), respectively. The modulus of elasticity of the mortar was determined from the axial compression test as 250 N/mm^2^ (CV 18%).

Table 3 presents the results of the tests on the bulk density and maximum density for the backfill materials used in the tests on buried vaults.

**Figure 8:**
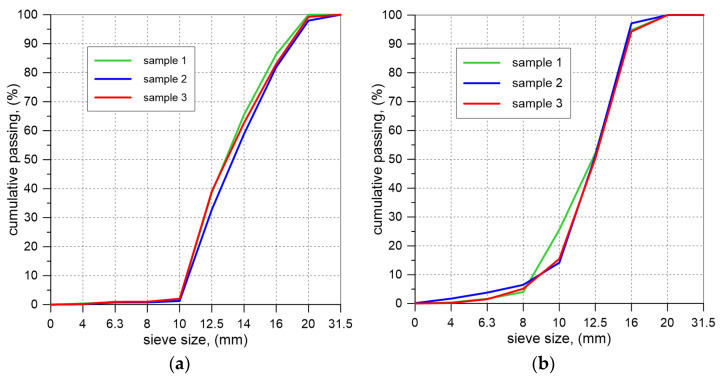
Particle size distribution curve for: (**a**) ECA; (**b**) granite.

For both backfill materials, the particle size distribution curve was plotted (Figure 8), and the grain size was determined. For ECA and granite aggregate, the grain sizes were 10/20 and 8/16, respectively. Additionally, the angle of repose was determined: 37° for ECA and 40° for the granite aggregate.

**Figure 9:**
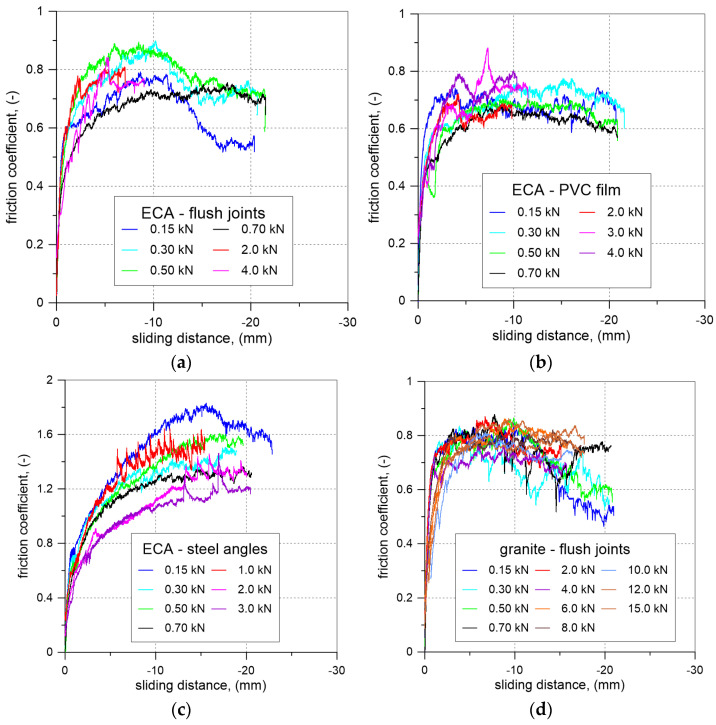

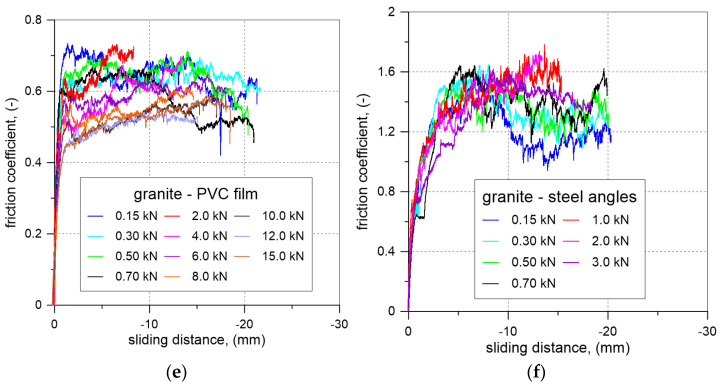
Results of the tests on friction coefficient between aggregate and masonry: (**a**) ECA—flush joints; (**b**) ECA—PVC film; (**c**) ECA—steel angles; (**d**) granite—flush joints; (**e**) granite—PVC film; (**f**) granite—steel angles.

**Figure 10:**
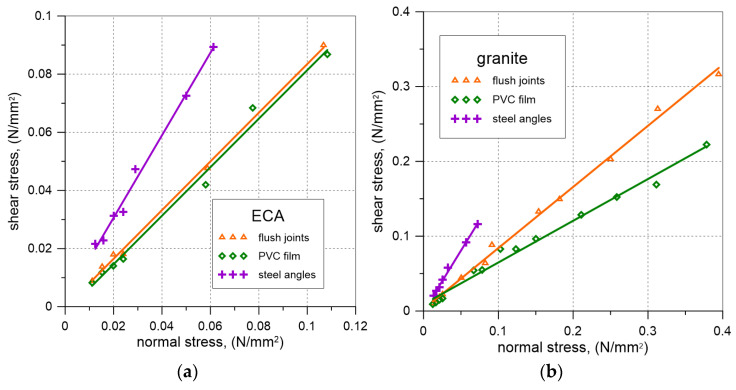
Results of the tests on friction coefficient—relation between normal and shear stress for different types of masonry finishing: (**a**) ECA; (**b**) granite.

Figure 9 illustrates the relationships between the friction coefficient (the ratio of normal stress to shear stress at the interface) and the sliding distance of the aggregate sample for different surface finishing methods of the masonry prism under varying vertical loads. Figure 10 presents the relationship between shear stress and normal stress at the interface between the aggregate and the masonry prism for both expanded clay aggregate (ECA) and granite, considering various surface finishing methods of the masonry prism.

The values of the friction coefficients obtained in the tests are summarized in Table 4.

**Figure 11:**
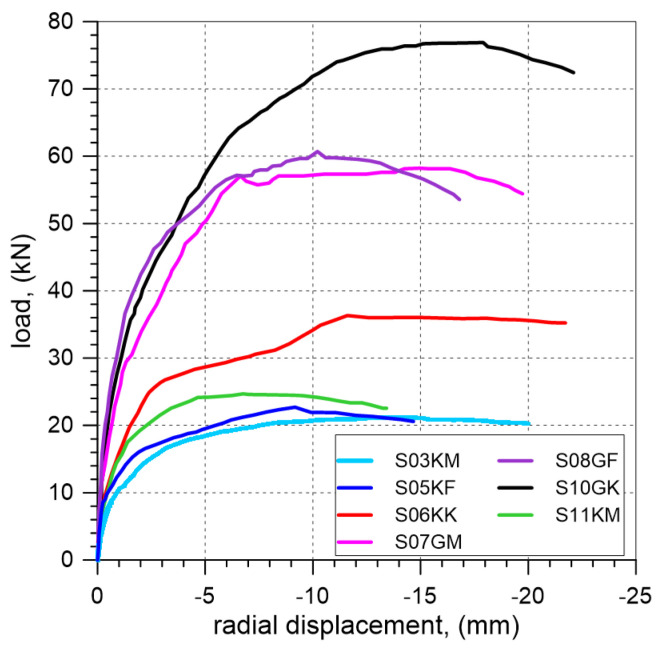
Results of the tests on masonry vaults with backfill (radial displacement-load curves) for specimens with different backfill material and various extrados finishing.

In the test on the vaults, the load, radial displacements of the vault, vertical displacements of the backfill, and horizontal displacements of the walls were measured. The relationships of load and radial displacement of brick course 12B (see Figure 1a) for all the tested vaults are presented in Figure 11.

**Figure 12:**
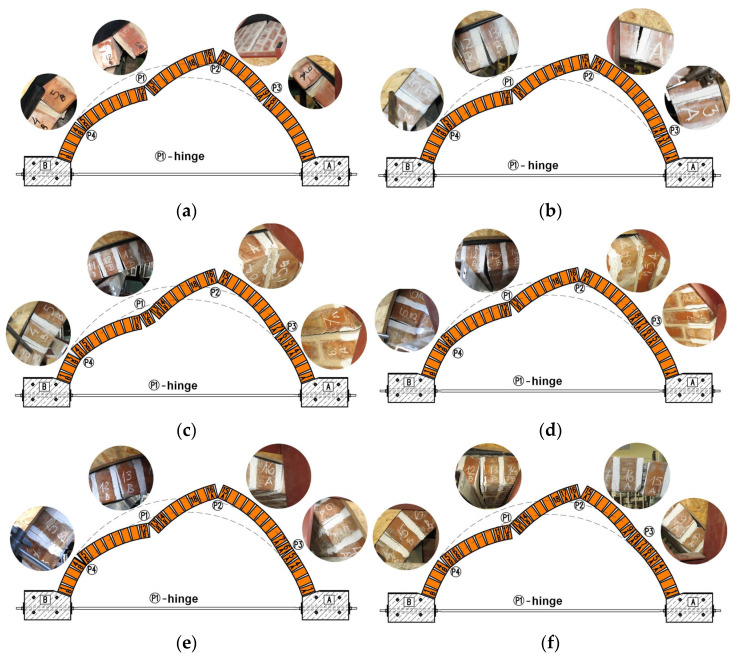
Failure modes obtained in the laboratory tests on masonry vaults with backfill: (**a**) S03KM; (**b**) S05KF; (**c**) S06KK; (**d**) S07GM; (**e**) S08GF; (**f**) S09GK.

The main objective of the tests on buried vaults was to determine the failure loads and observe the modes of failure of the tested specimens. All the tested vaults failed by the formation of a four-hinged mechanism (Figure 12). The obtained failure loads were dependent on the type of backfill material used and the method of extrados finishing, as summarized in Table 5.

### 3.2. Validation of the Model and Results of Numerical Simulation

**Figure 13:**
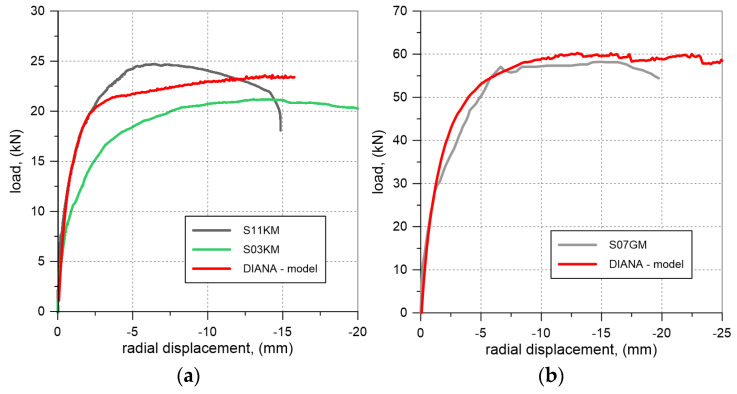
Comparison of load-radial displacement curves from laboratory tests and numerical analysis: (**a**) vaults with ECA backfill; (**b**) vaults with granite backfill.

To verify the accuracy of the FEM model, numerical simulations were conducted to analyze the behavior of vaults with ECA and granite aggregate backfill, with an unplastered extrados. The obtained results were compared with the data from the laboratory tests for vaults S03KM, S11KM, and S07GM. The failure loads for the vaults with ECA backfill were 21.2 kN and 24.8 kN, whereas the load capacity calculated using the FEM model was 23.3 kN. For the laboratory test of the vault with granite aggregate backfill, the failure load was 58.2 kN, while the numerical analysis predicted a failure load of 60.3 kN. The load-displacement curves obtained from the experiments and numerical simulations are presented in Figure 13.

**Figure 14:**
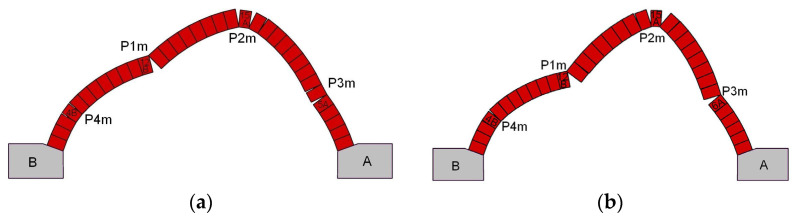
Selected failure modes obtained in FEM analysis for: (**a**) vault with ECA backfill; (**b**) vault with granite backfill (P1m to P4m notation of hinges).

Figure 14 presents the failure modes obtained from the FEM analysis of vaults with ECA and granite backfill. Both vaults failed by the formation of a four-hinged mechanism. In the model, the first hinge occurred under the force at the mortar joint 12B/13B. The second hinge, P2m, formed at the interfaces close to brick course 15A. The third hinge, P3m, appeared above brick course 5A, and finally the fourth hinge, P4m, formed close to the brick course 4B. The same sequence of hinge formation was observed in the experimental tests. The positions of the individual hinges differed slightly between the experimental results and the numerical simulation (see Figure 12a and Figure 14a). The difference lies in the location of the third hinge. The sequence of hinge formation in the FEM model of vault with granite backfill was the same as that for the element with ECA backfill and corresponded to the order observed in the experiment. The first hinge (P1m) formed at the extrados in the mortar joint between the masonry elements 12B and 13B. Cracks then developed in the mortar joints close to brick course 15A forming the second hinge, P2m. The third hinge, P3m, appeared between the brick courses 6A and 7A. The last hinge formed at the intrados in the mortar joints near the brick course 4B—hinge P4m. In this case, the positions of the hinges observed in the laboratory and those obtained in the numerical analysis were the same (see Figure 12d and Figure 14b).

**Figure 15:**
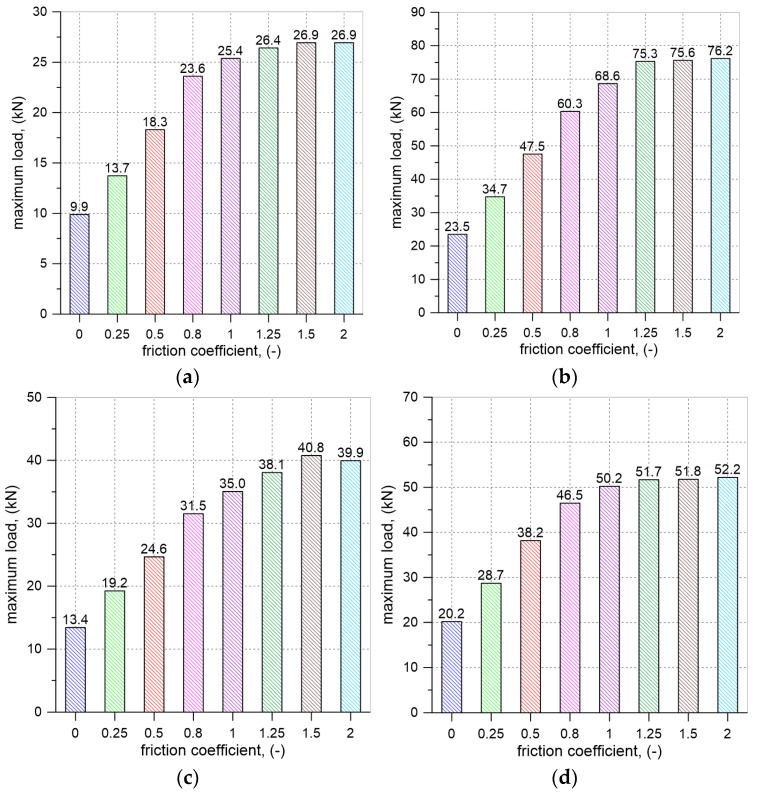

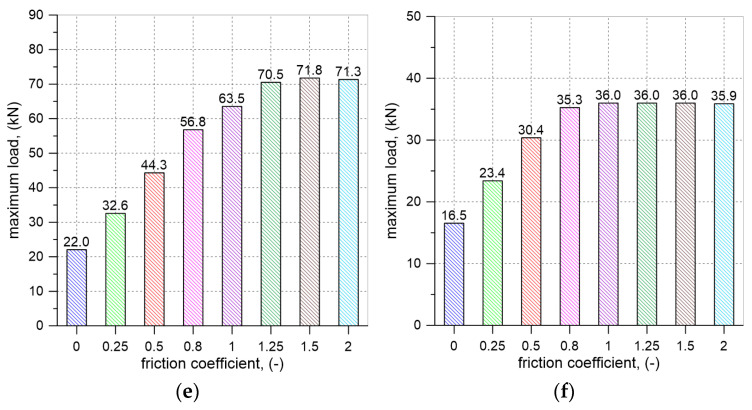
Results of FEM analysis. Maximum loads obtained in numerical analysis for different backfill materials and different friction coefficients: (**a**) ECA; (**b**) granite; (**c**) AM1; (**d**) AM2; (**e**) AM3; (**f**) AM4.

The validated model of buried vault was adopted for further parametric analyses aimed at investigating the influence of bulk density, internal friction angle of the backfill, and the friction coefficient between the backfill and the vault extrados on the load-bearing capacity of the buried vault. For the calculations, six different backfill materials were assumed (Table 2). In the numerical simulations, all analyzed vaults failed due to the formation of a four-hinged mechanism. The obtained failure loads depended on the chosen internal friction angle, bulk density of the backfill materials, and the friction coefficients assumed for the interface between the backfill and the extrados of the vault. The results of the computations (maximum loads) for the analyzed materials (ECA, Granite, AM1-AM4) are presented in Figure 15.

## 4. Discussion

Based on the results of laboratory tests on friction coefficient, it can be observed that the highest friction coefficient between the backfill material and the masonry prism, regardless of the aggregate used, was achieved in tests of specimens with a steel angle mounted on the surface of the masonry prism (Table 4). The presence of a PVC film between the aggregate and the masonry prism caused a reduction in the friction coefficients compared to the unplastered masonry prism for both tested materials. In the case of granite aggregate, this difference was noticeable, while for ECA, the decrease in the friction coefficient was minimal. Due to the small size of the sample in relation to the grain size of the tested aggregates, the results may have been affected by the limitations imposed by the steel box, which restricted the lateral deformations of the sample and the vertical movement of the aggregate particles.

At the laboratory, three vaults with unplastered extrados were tested, including two elements with ECA and one with granite aggregate. The highest load-bearing capacity was achieved for the vault with granite—58.2 kN, while the lowest was observed for the element filled with ECA—21.2 kN (S03KM) and 24.7 kN (S11KM). The increase in load-bearing capacity when using granite instead of ECA was 175% (S03KM) and 136% (S11KM). The failure modes of the individual elements were similar. During loading, four hinges (P1, P2, P3, and P4) formed (Figure 12). The position of the hinges in the different vaults varied slightly. In the case of vaults with an extrados covered by a PVC film, two elements were tested, one with ECA and one with granite. The failure loads for the vaults filled with ECA and granite were 22.7 kN and 60.7 kN, respectively. The increase in load-bearing capacity of the vault with granite compared to the one with ECA was 167%. In both elements, four hinges formed at maximum load (Figure 12). The vaults with steel angles mounted on the extrados failed at the load of 76.9 kN for the granite backfill and at 36.3 kN for the ECA backfill. The load-bearing capacity of the vault with the granite backfill increased by 112% compared to the one with ECA. The differences in the slope of the curves presented in Figure 11 indicate a lower stiffness of the specimens with ECA backfill when compared to the specimen with granite backfill.

**Figure 16:**
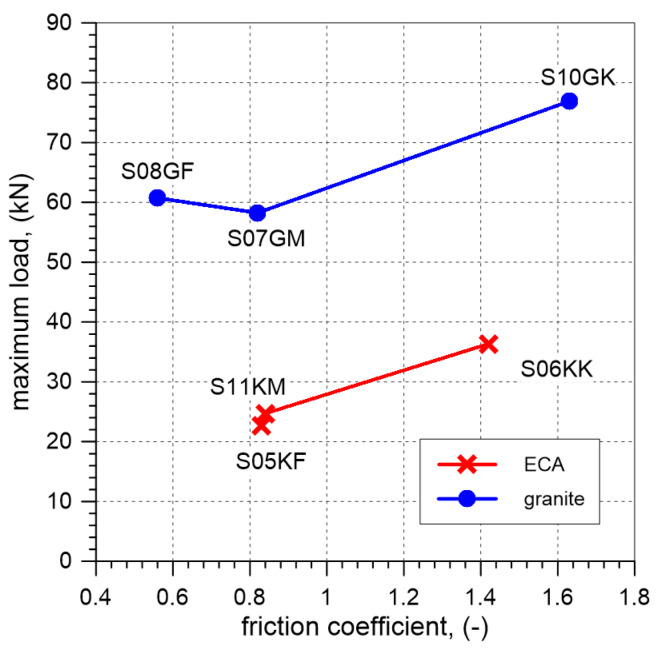
The results of laboratory test. Failure load vs. friction coefficient relations.

The use of a PVC film on the extrados of the vault had a negligible effect on the load-bearing capacity of the vault. In the case of the ECA backfill, the load-bearing capacity of the vault with the PVC film was approximately 7% higher compared to S03KM, but 8% lower than that of S11KM. For the granite backfill, the load-bearing capacity of the element with the PVC film was 4% higher. Therefore, based on the conducted tests, it cannot be conclusively determined whether the presence of PVC film between the backfill and masonry positively affects the load-bearing capacity of the vaults. The observed differences in load-bearing capacity may indicate a negligible impact of such a solution. The application of steel angles mounted on the extrados of the vaults resulted in an increase in the load-bearing capacity of the tested elements compared to the vaults without angles. The highest load-bearing capacity was achieved for S10GK. An increase in load-bearing capacity of vaults with steel angles compared to the elements with unplastered extrados were approximately: 47% for the ECA backfill and 32% for the granite backfill (Figure 16, Table 5).

The results of the laboratory tests conducted on the vaults indicate that the properties of the backfill materials significantly influence the load-bearing capacity of the buried vault. The highest load-bearing capacity was achieved for the vault with granite aggregate, a material characterized by the highest internal friction angle and bulk density. The lowest load-bearing capacity was observed for the specimen with ECA, a material with the lowest bulk density but a relatively high internal friction angle (Table 5). Due to the different properties of the materials used, it is difficult to definitively determine which of the properties, such as bulk density or internal friction angle, has a greater influence on the load-bearing capacity of the tested elements. In the tests different methods of finishing of the extrados of the vaults were also analyzed. Based on the results of these experiments, it is not possible to determine how the method of finishing of the extrados of the vault affect the failure load of the buried structure. To address these questions, numerical simulations were conducted.

**Figure 17:**
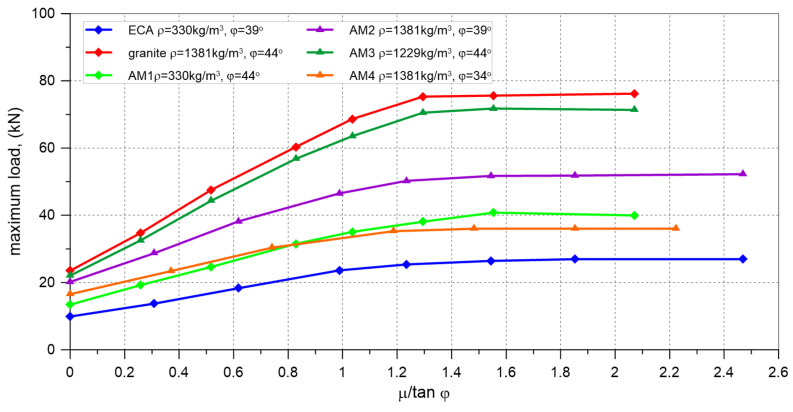
Results of FEM analysis. Maximum load vs. μ/tanφ curves.

**Figure 18:**
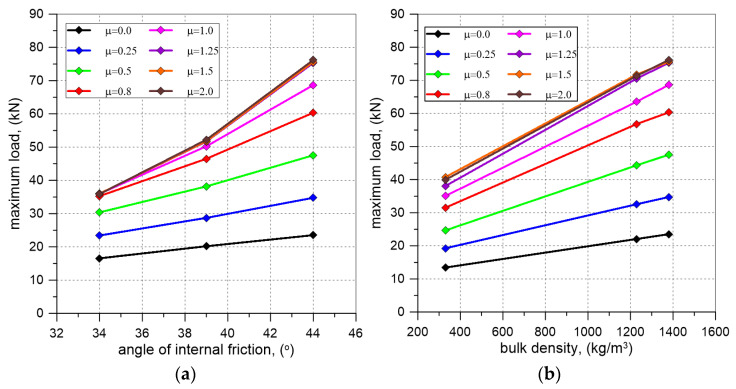
Results of FEM analysis: (**a**) maximum load vs. angle of internal friction of backfill material curves; (**b**) maximum load vs. bulk density of backfill material curves.

The results of numerical analyses indicate that the failure loads depend on the adopted internal friction angle (φ) and the bulk density of the backfill materials, and the friction coefficient (μ) between the backfill and the vault (Figure 15, Figure 17 and Figure 18).

Regardless of the assumed internal friction angle of the backfill material and its bulk density, an increase in the friction at the interface led to a higher failure load for the vault. The load capacities obtained in the simulations for the maximum friction coefficient values were at least twice as high as the failure loads obtained for the elements where the interface friction coefficient was assumed to be μ = 0. The greatest increases in failure load were observed for cases where the ratio μ/tanφ was less than 1.0. For μ/tanφ values greater than 1.5 (ECA, AM1, AM2), 1.3 (granite, AM3), and 1.2 (AM4), the load capacity obtained from the simulations stabilized at nearly constant levels. The greatest increase in the failure load, more than three times, was observed for the vault with granite. This clearly demonstrates the significant impact of method adopted for finishing of extrados on the load-bearing capacity of this type of structure.

The load-bearing capacity was also influenced by the properties of the backfill material itself, such as bulk density and internal friction angle. The highest load-bearing capacity was achieved for vaults with granite, which, among all the analyzed backfill materials, had the highest bulk density and internal friction angle. The lowest failure loads were obtained for the lightest material, expanded clay aggregate, which had a lower internal friction angle than granite but higher than the value assumed for the AM4 aggregate. For the remaining backfill materials (AM1, AM2, AM3, AM4), the computed load-bearing capacities were between the failure loads noticed for ECA and granite. When comparing the results obtained for granite and the backfill materials AM2 and AM3, it was found that changes in bulk density had a smaller impact on the load-bearing capacity of the vault with backfill than similar percentage changes in the internal friction angle. Decrease in the bulk density of the backfill material by approximately 11% led to a reduction in load-bearing capacity up to 7%. In contrast, a reduction in the internal friction angle of the backfill by about 11% caused a decrease in the failure load up to 31%.

Both the laboratory tests and numerical simulations indicate a significant impact of the internal friction angle, bulk density of the backfill materials, and the finishing method of the extrados of the vault on the load-bearing capacity of buried vaults.

## 5. Conclusions

This paper presents the outcomes of laboratory tests and numerical simulations of masonry vaults with backfill. The primary conclusions drawn from the study are as follows:

- The internal friction angle and bulk density of backfill materials significantly influence the load-bearing capacity of buried vaults. The vault with granite backfill exhibited a higher load capacity compared to the vault with ECA backfill. The bulk density and internal friction angle of granite aggregate were higher than those of ECA. This indicates that careful selection of backfill material is critical when designing and analyzing vaults in buildings.
- Laboratory tests and numerical simulations showed that increased friction at the backfill-vault interface led to higher failure loads. This finding is important for engineers considering different finishing techniques of the vault extrados to optimize the performance and stability of buried vaults.
- Changes in the internal friction angle of the backfill material had a greater impact on the load-bearing capacity of the buried vault than changes in bulk density. An 11% reduction in the internal friction angle caused up to a 31% decrease in load capacity, whereas the same reduction in bulk density led to only a 7% decrease.

It should be noted that the presented studies addressed a relatively limited number of potential scenarios. The results and conclusions drawn from these laboratory tests and numerical simulations may serve as guidance for engineers when analyzing buried vaults in buildings. However, it is important to remember that finding two identical vaults in existing structures is unlikely, if not impossible. Each such construction should be treated as unique, and comprehensive investigations should be conducted to assess the properties of the materials used, as well as the history of the structure, particularly previous repairs and reconstructions.

## Figures and Tables

**Table 1:** Tested specimens.

Notation	Backfill	Extrados Finishing Method
S03KM	ECA	Flush joints
S11KM	ECA	Flush joints
S05KF	ECA	PVC film
S06KK	ECA	Steel angles
S07GM	Granite	Flush joints
S08GF	Granite	PVC film
S09GK	Granite	Steel angles

**Table 2:** Properties of backfill materials used in FEM analysis.

Backfill Material	Angle of Internal Friction (°)	Bulk Density (kg/m^3^)
ECA	39	330
Granite	44	1381
AM1	44	330
AM2	39	1381
AM3	44	1229
AM4	34	1381

**Table 3:** Bulk density of aggregates and density for maximum compaction.

No	Bulk Density (kg/m^3^)		Density for Maximum Compaction (kg/m^3^)	
	ECA	Granite	ECA	Granite
1	293	1368	376	1674
2	302	1404	362	1620
3	304	1372	369	1627
Average	300	1381	369	1640

**Table 4:** Friction coefficient obtained in the tests.

	Backfill	ECA	Granite
Finishing			
Flush joints		0.84	0.82
PVC film		0.83	0.56
Steel angles		1.42	1.63

**Table 5:** Failure loads for tested vaults.

Extrados Finishing	Failure Load (kN)	
	ECA	Granite
Flush joints	21.2/24.7 *	58.2
PVC film	22.7	60.7
Steel angles	36.3	79.9

* results for specimens S03KM and S11KM.

## Data Availability

The original contributions presented in the study are included in the paper; further inquiries can be directed to the corresponding author.
